# Effect of Bariatric Surgery on Flow-Mediated Vasodilation as a Measure of Endothelial Function: A Systematic Review and Meta-Analysis

**DOI:** 10.3390/jcm11144054

**Published:** 2022-07-13

**Authors:** Tannaz Jamialahmadi, Mona Alidadi, Stephen L. Atkin, Matthew Kroh, Wael Almahmeed, Seyed Adel Moallem, Khalid Al-Rasadi, John H. Rodriguez, Raul D. Santos, Massimiliano Ruscica, Amirhossein Sahebkar

**Affiliations:** 1 Surgical Oncology Research Center, Mashhad University of Medical Sciences, Mashhad, Iran; jamiat931@gmail.com; 2 Department of Nutrition, Faculty of Medicine, Mashhad University of Medical Sciences, Mashhad, Iran; alidadimona@yahoo.com; 3 School of Postgraduate Studies and Research, RCSI Medical University of Bahrain, Busaiteen 15503, Bahrain; satkin@rcsi-mub.com; 4 Digestive Disease and Surgery Institute, Cleveland Clinic Lerner College of Medicine, Cleveland, OH 44195, USA; krohm@ccf.org; 5 Heart and Vascular Institute, Cleveland Clinic Abu Dhabi, Abu Dhabi P.O. Box 124140, United Arab Emirates; wmahmeed@gmail.com; 6 Department of Pharmacology and Toxicology, College of Pharmacy, Al-Zahraa University for Women, Karbala, Iraq; moallem@mums.ac.ir; 7 Department of Pharmacodynamics and Toxicology, School of Pharmacy, Mashhad University of Medical Sciences, Mashhad, Iran; 8 Medical Research Centre, Sultan Qaboos University, Muscat P.O. Box 373, Oman; k.alrasadi@gmail.com; 9 Cleveland Clinic Abu Dhabi, Al Maryah Island, Abu Dhabi P.O. Box 112412, United Arab Emirates; rodrigj2@clevelandclinicabudhabi.ae; 10 Lipid Clinic Heart Institute (Incor), University of São Paulo, Medical School Hospital, São Paulo 01000, Brazil; rauldsf@gmail.com; 11 Department of Pharmacological and Biomolecular Sciences, Università degli Studi di Milano, 20100 Milan, Italy; massimiliano.ruscica@unimi.it; 12 Applied Biomedical Research Center, Mashhad University of Medical Sciences, Mashhad 9177948564, Iran; 13 Biotechnology Research Center, Pharmaceutical Technology Institute, Mashhad University of Medical Sciences, Mashhad 9177948954, Iran; 14 Department of Biotechnology, School of Pharmacy, Mashhad University of Medical Sciences, Mashhad 9177948954, Iran

**Keywords:** bariatric surgery, flow-mediated vasodilation, body mass index, endothelial function, meta-analysis

## Abstract

Objectives. Flow mediated vasodilation (FMD) is a marker of endothelial function and its decline is related to increased cardiovascular risk. This systematic review and meta-analysis evaluated the impact of bariatric surgery on FMD. Materials and methods. A systematic literature search in PubMed, Scopus, Embase, and Web of Science was performed to 1 May 2021. Meta-analysis was performed using Comprehensive Meta-Analysis (CMA) V2 software. All types of bariatric surgery were considered, with the inclusion that FMD had to have been tested before and after the surgical procedure. Meta-analysis was carried out using a random-effects model and the generic inverse variance approach. The leave-one-out approach was used for sensitivity analysis. To assess metabolic parameter confounders, a weighted random-effects meta-regression was used. Results. A meta-analysis and a systematic review of 23 studies (n = 891 individuals) demonstrated improvement in FMD following bariatric surgery (weighted mean difference (WMD): 5.867, 95% CI: 4.481, 7.252, *p* < 0.001; I^2^: 96.70). Iteratively removing each item in the meta-analysis did not result in a significant alteration in the pooled estimate of effect size. There was an improvement in FMD for up to 6 months following bariatric surgery in a meta-analysis from 7 trials that included 356 subjects (WMD: 5.248, 95% CI: 2.361, 8.135, *p* < 0.001; I^2^: 98.18). The meta-analysis from 9 trials (n = 414 subjects) showed an improvement in FMD 6 to 12 months after bariatric surgery (WMD: 5.451, 95% CI: 3.316, 7.587, *p* < 0.001; I^2^: 94.18). The meta-analysis from 10 trials (n = 414 subjects) demonstrated an improvement in FMD 12 months after bariatric surgery (WMD: 2.401, 95% CI: 0.944, 3.859, *p* = 0.001; I^2^: 88.35). Random-effects meta-regression did not show any association between the alteration in FMD and percent body mass index (BMI) change (slope: 0.0258; 95% CI: −0.323, 0.375; *p* = 0.884), or changes in blood pressure; however, there was an association between the changes in FMD and the duration of follow-up (slope: −0.106; 95% CI: −0.205, −0.008; *p* = 0.033) with greater changes in FMD after 12 months. Conclusions. Bariatric surgery significantly improved FMD that increased with time, and the resultant improvement in endothelial function was independent of weight loss or a reduction in blood pressure.

## 1. Introduction

Obesity is linked to an enhanced risk of cardiovascular disease and mortality [1]. Long-term weight loss maintenance with prevention and remission of hypertension, type 2 diabetes and dyslipidemia following Roux-en-Y gastric bypass has been shown [2], with a reduction in cardiovascular disease risk [3]. Bariatric surgery has been shown to increase life expectancy in individuals with elevated cardiovascular risk [4]. Endothelial dysfunction is an important factor in cardiovascular disease development and progression [5,6,7], and its clinical assessment has been proposed as a surrogate marker for cardiovascular risk stratification and disease prognosis [8,9,10,11,12]. Functional clinical evaluation of endothelial dysfunction can be determined noninvasively by flow mediated vasodilation (FMD) that represents a nitric oxide (NO)-mediated dilatation of conduit arteries, which is endothelium-dependent, through an induced increase in blood flow and shear stress [13].

Weight loss improves FMD [14] and it has been reported that bariatric surgery may improve FMD by improving vasculature remodeling in a wide spectrum of subjects with elevated cardiovascular risk [15]. Differential arterial responses to weight reduction surgery have been reported based on metabolic status, highlighting heterogeneity in physiological responses to weight loss and changes in adiposity, and probable activation of separate pathways in clinical subgroups [16]. However, FMD responses following bariatric surgery may be unchanged [17]. 

The objective of this comprehensive systematic review and meta-analysis of clinical trials was to evaluate the impact of metabolic surgery on FMD, evaluating the consistency of the effect with the duration of follow-up after the surgical procedure. The effect of weight reduction and changes in blood pressure on the FMD response were also tested. 

## 2. Methods

### 2.1. Search Strategy

The 2009 preferred reporting items for systematic reviews and meta-analysis (PRISMA) guidelines were utilized to compile this systematic review and meta-analysis [18]. Till May 1st, 2021, Scopus, PubMed, Embase and Web of Science were searched using the following keywords in titles and abstracts: (“bariatric surgery” OR gastrectom* OR gastroplast* OR “Roux-en-Y” OR “gastric bypass” OR “biliopancreatic diversion” OR “duodenal switch” OR “gastrointestinal diversion” OR “weight loss surgery” OR gastroenterostom* OR “jejunoileal bypass” OR “obesity surgery” OR “weight-loss surgery” OR “sleeve surgery” OR “bariatric procedure” OR “metabolic surgery” OR “gastric band”) AND (FMD OR bFMD OR fFMD OR “flow mediated dilation” OR “flow-mediated dilation” OR “flow mediated dilatation” OR “flow-mediated dilatation” OR “flow mediated vasodilation” OR “flow-mediated vasodilation” OR “flow mediated vasodilatation” OR “flow-mediated vasodilatation). The search strategy is shown in the Supplemental Material.

### 2.2. Study Selection

Only original publications written in English that were peer-reviewed were included. This study considered any type of bariatric surgery. To be considered for inclusion, publications must have reported FMD prior to and following surgery. Animal studies, abstract-only publications, non-English research, duplicate research, reviews, case reports, meta-analyses, comments, letters, and studies with an absence of outcomes, and no surgical intervention were excluded.

### 2.3. Data Extraction 

After removing duplicate studies, two blinded and independent reviewers (TJ, MA) were chosen. For eligibility, the titles and abstracts of the publications were examined. The full text of the included papers was gathered for additional review. If the same organization and/or authors found two (or more) publications on the same study objective, the one with the larger sample size was included. Discussion and consensus were used to resolve disagreements. The following data were extracted from relevant studies: (1) the identity of the first author, (2) the year of publication, (3) the study design, (4) the surgery type, (5) the length of follow-up, (7) patient characteristics, and (8) clinical outcomes.

### 2.4. Quality Assessment

The quality of the studies that were included in this systematic review and meta-analysis was independently estimated by two reviewers (TJ, MA) using the Newcastle-Ottawa Scale (NOS) [19,20]. The NOS includes: (1) the selection of the patients in the studies (4 items), (2) the determination of the exposure (3 items) in case-control studies or outcome of interest in cohort studies and (3) the comparability of the studied populations (one item).

### 2.5. Quantitative Data Synthesis

Comprehensive Meta-Analysis (CMA) V2 software was used for the meta-analysis (Biostat, NJ) [21]. For continuous outcomes, the weighted mean difference (WMD) with associated confidence intervals was calculated. For each relevant outcome, means, standard deviations as well as sample sizes, were acquired from each group to calculate weighted mean differences (WMDs). The overall estimate of effect size was calculated using a random effects meta-analysis. A random-effects model (using DerSimonian-Laird method) and the general inverse variance weighting technique were employed to account for heterogeneity of publications in terms of study design, features of the populations and treatment duration [18]. The mean and standard deviation values were calculated using the method described by Hozo et al. [22] if the outcome measures were reported in median and interquartile range (or 95% confidence intervals [CI]). When only standard error of the mean (SEM) was supplied, SD was determined using the following formula: SD = SEM × sqrt (n), where n denotes the number of participants. To examine the effect of each study on the overall effect size, a sensitivity analysis through the leave-one-out strategy was used [23]: sensitivity analyses, where one study is excluded at a time and the impact of removing each of the studies, is evaluated on the summary results and the between-study heterogeneity.

### 2.6. Meta-Regression

A random-effect meta-regression model was used to explore the relationship between BMI, SBP and DBP changes, as well as follow-up length after surgery, and the estimated effect size. 

### 2.7. GRADE Scoring

We used the Grade of Recommendations, Assessment, Development, and Evaluation (GRADE) approach to assess the strength of evidence for each outcome [24]. To summarize the findings for each outcome, the GRADEpro GDT software was used. We assigned four points to each outcome and then evaluated factors that reduced the quality of the evidence. For each outcome, points were reduced based on the presence of the following: the overall risk of bias (RoB) for each study, inconsistency (significant heterogeneity), indirectness (significant differences in the population, comparisons, and outcomes), imprecision (the size of the cohort, width and significance of the confidence intervals (CIs). As a result, we classified the evidence into four groups depending on the aggregate GRADE ratings for each intervention: high-grade evidence (at least 4 points), moderate grade evidence (3 points), low-grade evidence (2 points) and very low-grade evidence (1 point) (Table 1).

### 2.8. Publication Bias

To investigate the existence of publication bias in the meta-analysis, the funnel plot was used. Furthermore, Egger’s weighted regression and Begg’s rank correlation tests evaluated publication bias. When there was visual evidence of funnel plot asymmetry, the “trim and fill” method was employed to insert potentially missing publications. In the case of a significant result, the number of potentially missing studies required to make the *p*-value non-significant was calculated using the “fail-safe N” approach, which is yet another example of publication bias [25].

## 3. Results

**Figure 1:**
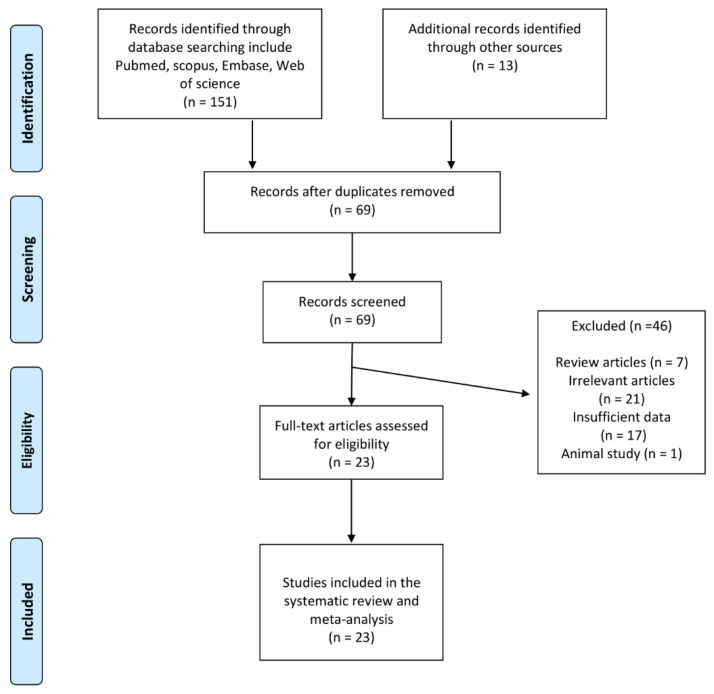
Flow chart of identified and included publications into meta-analysis.

A comprehensive database search yielded 164 publications, 95 of which were excluded after the title and abstract review. Of the 69 articles screened in full text, 46 studies were excluded (7 papers were reviews, 21 publications were excluded because they did not meet the inclusion criteria, 17 studies did not report enough data and one was an animal study). As a result, 23 prospective observational studies evaluating FMD following bariatric surgery were considered (Table 2). Figure 1 shows the study selection procedure.

Endothelial function was assessed in all individuals using brachial artery ultrasonography. Images of the brachial arteries were taken using a high-resolution (7.5-MHz) transducer 3 to 5 cm above the right antecubital fossa. To obstruct arterial flow, a blood pressure cuff was inflated to 50 mm Hg suprasystolic for five minutes. The blood pressure cuff was then deflated, and the brachial artery was reimaged one minute later, when maximal vasodilation occurs. FMD is caculated as a percentage using the following formula: (maximum diameter baseline diameter)/baseline diameter × 100. Continuous gated electrocardiography was used to take measurements from the near intimal interface to the far wall at end-systole [26].

### 3.1. Quality Assessment of the Included Studies

Most of the selected publications revealed a lack of representativeness of the cases, case definition information, control selection, as well as control definition. Since most of the studies lacked a control group, they were not evaluated for comparability, same method of ascertainment, or non-response rate. Eventually, all of the considered publications met the ascertainment of exposure criteria only. The quality of the included publications is assessed in Table 3.

### 3.2. Publication Bias

**Figure 2:**
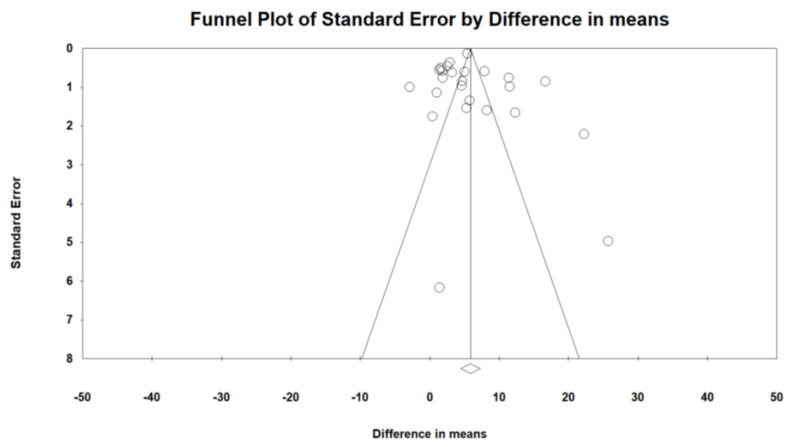
Funnel plot detailing publication bias in the publications describing the effect of bariatric surgery on FMD.

Evaluation for bias using Egger’s (intercept = 0.700, standard error = 1.62; 95% CI = −2.66, 4.06, t = 0.430, df = 23, two-tailed *p* = 0.670) and Begg’s test (Kendall’s Tau with continuity correction = 0.25, z = 1.751, two-tailed *p*-value = 0.079) suggested that there was no publication bias in the meta-analysis demonstrating bariatric surgery’s impact on FMD. Trim and fill correction identified two “missing” studies. In accordance with the “fail-safe N” test, 8556 missing papers would be required to lower the effect size to a non-significant (*p* < 0.001) level (Figure 2).

### 3.3. Impact of Bariatric Surgery on FMD

**Figure 3:**
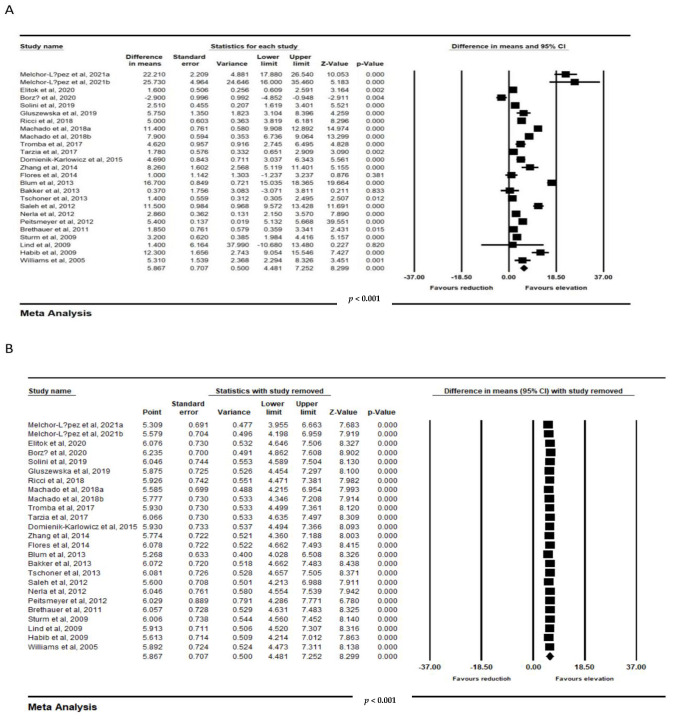
(**A**). Forest plot displaying standardized mean difference and 95% confidence intervals showing the consequence of bariatric surgery on FMD. (**B**). Leave-one-out sensitivity analyses indicating the consequence of bariatric surgery on FMD (891 subjects).

Meta-analysis from 23 trials including 891 individuals confirmed a significant improvement in FMD following bariatric surgery (WMD: 5.867, 95% CI: 4.481, 7.252, *p* < 0.001; I^2^: 96.70) (Figure 3A). Iteratively removing each item in the meta-analysis did not result in a significant alteration in the pooled estimate of the effect size (Figure 3B).

### 3.4. Impact of Bariatric Surgery on FMD at Different Follow Up Time Points 

For studies which evaluated the impact of bariatric surgery at multiple time points, we repeated the same test at different time periods. There were significant improvements in FMD after bariatric surgery at three time points. (A: <6 months; B: ≥6 months to <12 months; and C: ≥12 months).

**Figure 4:**
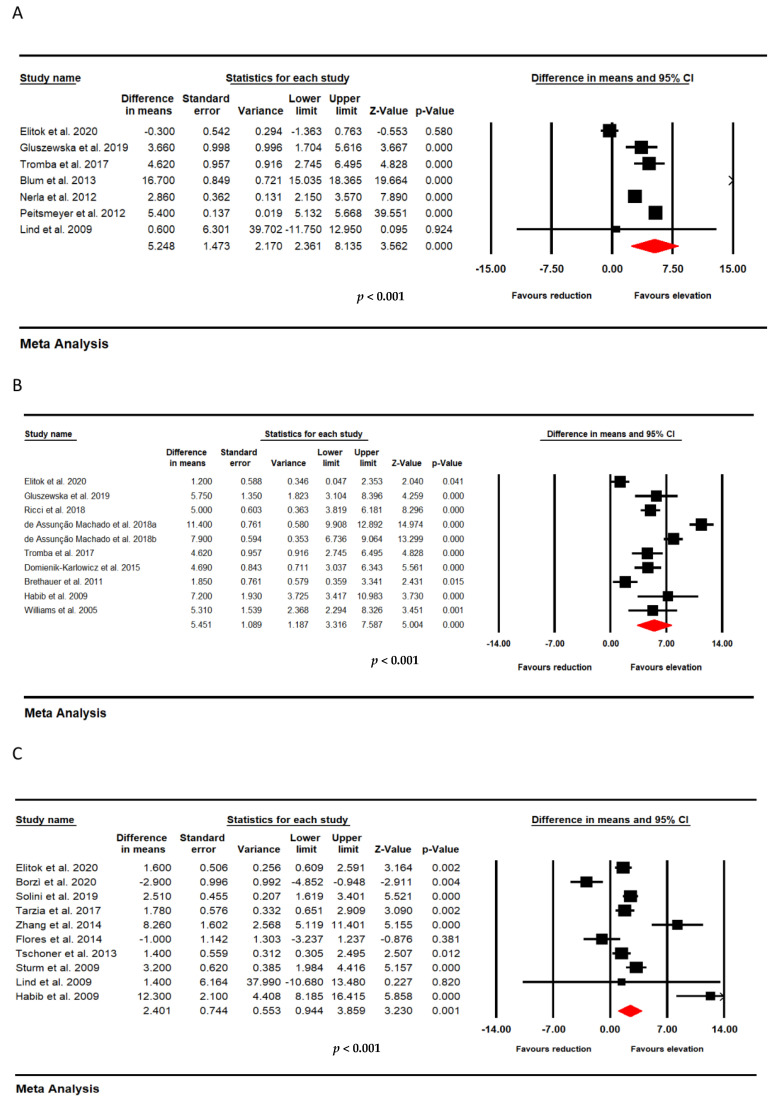
Effect of bariatric surgery on FMD at different follow up time point. (**A**): <6 months (356 subjects); (**B**): ≥6 months < 12 months (414 subjects); and (**C**): ≥12 months (414 subjects).

In time point A, a significant improvement in FMD following bariatric surgery was demonstrated in a meta-analysis of 7 trials that included 356 subjects (WMD: 5.248, 95% CI: 2.361, 8.135, *p* < 0.001; I^2^: 98.18) (Figure 4A). In time point B, from 9 trials that included 414 subjects, a significant increase in FMD following bariatric surgery was observed (WMD: 5.451, 95% CI: 3.316, 7.587, *p* < 0.001; I^2^: 94.18) (Figure 4B). In time point C, a meta-analysis of 10 studies that included 414 subjects confirmed a significant increase in FMD after bariatric surgery (WMD: 2.401, 95% CI: 0.944, 3.859, *p* = 0.001; I^2^: 88.35) (Figure 4C).

### 3.5. Meta-Regression

**Figure 5:**
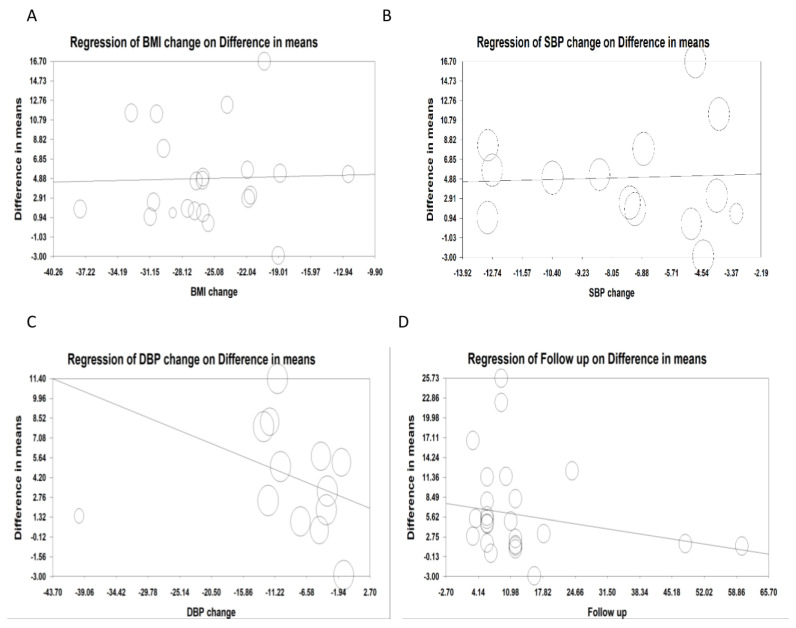
Random-effects meta-regression for evaluating the effect of: (**A**). BMI change (in 842 subjects); (**B**). SBP change (in 500 subjects); (**C**): DBP change (in 500 subjects); (**D**): follow up duration (in 891 subjects).

Random-effects meta-regression was used to analyze the effect of various variables on the FMD-reducing effect of bariatric surgery. The results did not show any association between the changes in FMD and the percent BMI change (slope: 0.0258; 95% CI: −0.323, 0.375; *p* = 0.884) in 842 subjects. The results did not show any association between the changes in FMD and the percent SBP change (slope: 0.064; 95% CI: −0.717, 0.846; *p* = 0.870) in 500 subjects, nor any change between FMD and percent DBP change (slope: −0.203; 95% CI: −0.512, 0.105; *p*= 0.197) in 500 subjects. There was a significant association between the change in FMD and duration of follow-up (slope: −0.106; 95% CI: −0.205, −0.008; *p* = 0.033) in 891 subjects (Figure 5A–D)**.**

### 3.6. Subgroup Analysis

**Figure 6:**
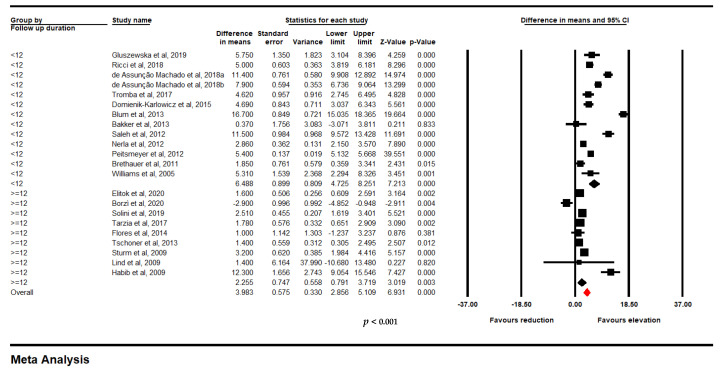
Subgroup analysis based on the follow up duration.

A subgroup analysis was also performed based on change in FMD and the duration of follow up (≥12 months in 251 subjects and <12 months in 640 subject). Bariatric surgery was associated with the maintenance of the increased FMD according to follow up duration (WMD: 7.789, 95% CI: 5.958, 9.620, *p* < 0.001; I^2^: 96.87 for <12 months and WMD: 2.785, 95% CI: 1.273, 4.297, *p* < 0.001; I^2^: 89.43 for ≥ 12 months (Figure 6).

## 4. Discussion

This comprehensive systematic review and meta-analysis has shown that there was an overall beneficial effect of bariatric surgery on FMD over a period of 6 to 12 months with an enhanced benefit after 12 months. In addition, the effects were not associated with the degree of weight loss nor changes in arterial blood pressure.

In current clinical practice, CVD risk is assessed by identifying and measuring identified risk factors such as diabetes, hypertension, dyslipidemia and smoking, and using the composite in a risk calculator [50]. However, there is significant variation in response to risk factors and medications. Nontraditional and unknown risk factors may potentially play a significant influence in atherosclerosis [51], and this may lead to bias and an under or overestimation of cardiovascular risk. However, a functional importance of atherogenesis can be determined by assessing endothelial function. FMD is a noninvasive peripheral endothelial function test that is feasible and useful in the stratification of cardiovascular risk [52], and therefore highly attractive, although its use in routine clinical practice is currently limited.

The results reported here are in accordance with a limited meta-analysis of eight studies that reported an improvement in FMD after surgery [15] within a 12-month period. It is well recognized that cardiovascular risk parameters improve following bariatric surgery [3,53], and one of these parameters would be the improvement in endothelial cell function that is reflected in the increase in FMD [13]. Furthermore, in another meta-analysis evaluating the impact of bariatric surgery on cardiovascular risk, we showed that pulse wave velocity (PWV), as a measure of arterial stiffness, favorably predicts subsequent cardiovascular outcomes [12]. In this study, the improvement in FMD was present at 6 months with enhanced improvement after 12 months. However, the studies evaluated did not specify when the initial improvement in FMD occurred. In one study, a significant alteration in metabolic status was reported after 10 days, including systolic blood pressure, glucose, high density lipoprotein cholesterol, leptin, insulin and insulin resistance, however, changes in FMD were only seen after 6 months [16]. On the other hand, long-term follow-up of patients’ weight reveals significant inter-individual variability, i.e., ongoing weight reduction, weight stabilization, or weight regain [54]. In terms of the influence of fat gain on endothelial function, weight increase resulted in lower FMD after 8 weeks compared to patients who don’t gain weight but returned to baseline levels if normal weight was restored [55]. Furthermore, the degradation of endothelial function was substantially associated with an increase in visceral fat but not subcutaneous fat.

Random-effects meta-regression did not show any significant association between the changes in FMD and percent BMI change. This result was in contrast to the hypothesis that endothelial improvement may reflect increasing weight loss [15,16]. Surprisingly, there is no conclusive evidence that non-surgical weight loss in patients with obesity is associated with a lower risk of cardiovascular events [56]. These findings suggest that endothelial function may be improved via weight-independent processes such as endocrine and incretin-mediated effects as well as the improved inflammatory status [47,57]. It is suggested that positive metabolic and cardiovascular effects of bariatric surgery may also be due to changes in intestinal physiology rather than to weight loss alone. The gut hormone GLP-1, which rises immediately after surgery and restores glycemic homeostasis, may have a role in post-surgery cardiovascular protection [58].

Borzi et al. suggested that a slight but significant increase in cardiovascular events occurs in the first months following surgery, when compared with a non-surgical control group [17]. However, because cardiovascular events follow vascular disease progression that develop over time, it is also reasonable to assume that reducing obesity-related risk factors will also reflect in a reduction in cardiovascular events over time. The findings of an early cardiovascular increase in the first 6 months of surgery may be due to a premature evaluation on too few patients, suggesting that prospective studies are needed to clarify this.

Previous smaller meta-analyses suggested that BMI and body weight reductions were the most important predictors of FMD improvement [15]. The random-effects meta-regression analysis here revealed no significant relation between the changes in FMD and systolic or diastolic blood pressure. It is well-recognized that decreases in blood pressure following bariatric surgery [49] may not contribute to the mechanisms underlying the improvement in FMD seen following bariatric surgery.

A major strength of this study is the use of a meta-analysis with a larger population size compared to the individual studies that were small and, in some cases, underpowered to determine if bariatric surgery had an impact on FMD. Limitations of the meta-analysis performed here include that the majority of the chosen studies showed a lack of representativeness of the cases, the selection of controls differed, and case definition information varied. Most of the publications lacked a control group and were not evaluated for comparability, non-response rate or same method of ascertainment. Concerns about the reproducibility of FMD have been raised [13], though when standardized protocols are used, then highly reliable FMD measurements are obtained. However, this is unlikely to be the case between these studies, and a risk of reporting bias cannot be excluded. This may have been reflected by the high heterogeneity seen among the different studies tested. Several trials had a modest population size and a small number of individuals; nonetheless, according to an earlier study with a large sample size, the population assessed was sufficiently powerful. It was not possible to determine if one form of bariatric surgery was more effective than another for FMD changes. Finally, it should be noted that the primary endpoint of the studies included here were not the effect of bariatric surgery on endothelial function. The present study also looked at FMD in subjects longitudinally who had either no weight loss or subsequent weight gain, to determine if the improvement in FMD was maintained. The duration of follow-up differed between studies that may have contributed to heterogeneity seen in our findings. This study was not registered on PROSPERO prior to undertaking the evaluation, with the concern that this may introduce potential bias to the review.

In conclusion, this meta-analysis showed that bariatric surgery significantly improved FMD, and that this increased with time. The resultant improvement in endothelial function was independent of weight loss or a reduction in blood pressure. This evidence supports the use of bariatric surgery as a therapy with the potential to reduce CV morbidity/mortality, particularly in individuals with a high obesity-related CV risk. More large, randomized trials comparing the CV effects of various bariatric surgery methods to appropriate medical therapy are needed. 

## Figures and Tables

**Table 1:** GRADE assessment.

Effect of Bariatric Surgery on Flow-Mediated Vasodilation						
**Patient or population:** Patients with obesity **Setting:** - **Intervention:** Bariatric surgery **Comparison:** -						
Outcome № of participants (studies)	Relative effect (95% CI)	**Anticipated absolute effects (95% CI)**			Certainty	What happens
				**Difference**		
FMD assessed with: B-mode ultrasound № of participants: (23 observational studies)	-		-	MD **5.867%** (CI: 4.481 to 7.252)	⨁◯◯◯ Very low	
* **The risk in the intervention group** (and its 95% confidence interval) is based on the assumed risk in the comparison group and the relative effect of the intervention (and its 95% CI). **CI:** confidence interval; **MD:** mean difference						
**GRADE Working Group grades of evidence** **High certainty:** we are very confident that the true effect lies close to that of the estimate of the effect. **Moderate certainty:** we are moderately confident in the effect estimate: the true effect is likely to be close to the estimate of the effect, but there is a possibility that it is substantially different. **Low certainty:** our confidence in the effect estimate is limited: the true effect may be substantially different from the estimate of the effect. **Very low certainty:** we have very little confidence in the effect estimate: the true effect is likely to be substantially different from the estimate of effect.						

Explanations: Most of studies have an unclear risk of bias across five or more domains. Thus, we downgraded one level. A considerable level of heterogeneity. Therefore, we downgraded one level. There was publication bias in the studies.

**Table 2:** Characteristics of studies measuring FMD.

Study, Year	Study Design	Follow-Up	Treatment	Control	Clinical Outcome		Patients	No. of Patients
					Brachial FMD	% BMI Change		
Williams et al., 2005 [27]	Longitudinal study	At 6 months or after 10% weight loss	Gastric banding	-	Unchanged		Morbidly obese patients	6
Habib et al., 2009 [28]	Prospective study	6 months 24 months	RYGB	-	Significant increase in FMD levels		Obese patients	45 28
Lind et al., 2009 [29]	Prospective observational study	1 months 12 months	RYGB	Non-obese controls matched for age and gender	Unchanged		Obese patients	19
Sturm et al., 2009 [30]	Prospective study	18 months	LAGB or RYGB		Significant increase in FMD levels compared with baseline		Obese patients	25
Tschoner et al., 2013 [31]	Prospective study	5 years	Swedish adjustable gastric banding (SAGB) or gastric bypass (GBP)	-	Significant increase in FMD levels compared with baseline		Obese patients	36
Brethauer et al., 2011 [32]	Prospective longitudinal study	6 months	LRYGB	-	Unchanged		Obese patients	15
Peitsmeyer et al., 2012 [33]	Prospective observational study	Mean = 3.6 months	RYGB	-	Significant increase in FMD levels		Morbidly obese patients	46
Nerla et al., 2012 [34] Tarzia et al., 2017 [35]	Prospective observational study	3 months Mean = 4.5 years	RYGB or biliopancreatic diversion	Comparable obese controls without any evidence of cardiovascular disease -	Significant increase in FMD levels compared with baseline and control group Significant increase in FMD levels	−37.73	Obese patients without any evidence of cardiovascular disease	50 19
Saleh et al., 2012 [36]	Prospective cohort study	Mean = 10 months	RYGB	-	Significant increase in FMD levels		Morbidly obese patients	47
Bakker et al., 2013 [37]	Case-control study	Mean = 204 days	RYGB or gastric banding	Obese patients with obstructive sleep apnea underwent CPAP	Unchanged	−25.64	Obese patients with obstructive sleep apnea	12
Blum et al., 2013 [38]	Prospective study	3 months	SG or gastric banding	-	Significant increase in FMD levels	−20.36	Obese patients	102
Flores et al., 2014 [39]	Prospective study	12 months	LRYGB or SG	-	Unchanged	−31.11	Hypertensive obese patients	33
Zhang et al., 2014 [40]	Prospective observational study	12 months	LRYGB	-	Significant increase in FMD levels		Hypertensive patients with type 2 diabetes	9
Domienik-Karlowicz et al., 2015 [41]	Prospective study	6 months	RYGB	Healthy women	Significant increase in FMD levels	−26.20	Morbidly obese premenopausal women with metabolic syndrome	40
Tromba et al., 2017 [42]	Prospective observational study	3 months 6 months	SG	-	Significant increase in FMD levels	−26.77	Obese patients	45
Machado et al., 2018 [43]	Case-control study	6 months	RYGB	-	Significant increase in FMD levels in both groups	−30.52 −29.86	Obese patients without obstructive sleep apnea Obese patients with obstructive sleep apnea	28 28
Ricci et al., 2018 [44]	Prospective observational study	10–12 months	SG	-	Significant increase in FMD levels	−26.15	Obese patients	110
Gluszewska et al., 2019 [45]	Prospective observational study	10 days 6 months	LRYGB or SG	-	Significant increase in FMD levels at 6 months	−21.94	Obese patients	71
Solini et al., 2019 [46]	Prospective observational study	12 months	RYGB	-	Significant increase in FMD levels	−30.80	Obese non-diabetic patients	25
Borzì et al., 2020 [17]	Case-control study	Mean = 16 months	AGB, RYGB or biliopancreatic diversions	Obese individuals who underwent medical nutrition treatment	Significant increase in FMD levels	−19.04	Obese patients	17
Elitok et al., 2020 [47]	Prospective observational study	3 months 6 months 9 months 12 months	RYGB	-	Significant increase in FMD levels at 6, 9 and 12 months	−26.92	Morbidly obese patients	23
Melchor-López et al., 2021 [48]	Case-control study	9 months	RYGB or SG	-	Significant increase in FMD levels in patients who had 2-fold increase in FMD		Obese patients, ≥2-fold increase in FMD Obese patients, ≤2-fold increase in FMD	25 15

**Table 3:** Quality of bias assessment of the included publication in accordance with the Newcastle-Ottawa scale.

Study	Selection				Comparability	Exposure		
	Case Definition	Representativeness of the Cases	Selection of Controls	Definition of Controls	Comparability of Cases and Controls	Ascertainment of Exposure	Same Method of Ascertainment	Non-Response Rate
Bakker et al., 2013 [37]	*	-	-	-	*	*	-	-
Blum et al., 2013 [38]	-	-	-	-	-	*	-	-
Borzì et al., 2020 [17]	-	-	-	*	-	*	-	-
Brethauer et al., 2011 [32]	-	-	-	-	-	*	-	-
Machado et al., 2018 [43]	*	-	-	*	*	*	*	-
Domienik-Karłowicz et al., 2015 [41]	-	-	-	-	*	*	-	-
Elitok et al., 2020 [47]	-	-	-	-	-	*	-	-
Flores et al., 2014 [39]	-	-	-	-	-	*	-	-
Gluszewska et al., 2019 [45]	-	-	-	-	-	*	-	-
Habib et al., 2009 [28]	-	*	-	-	-	*	-	-
Lind et al., 2009 [29]	-	-	-	*	*	*	-	-
Melchor-López et al., 2021 [48]	-	-	-	-	*	*	*	-
Nerla et al., 2012 [34]	-	*	-	-	**	*	*	-
Peitsmeyer et al., 2012 [33]	-	*	-	-	-	*	-	-
Ricci et al., 2018 [44]	-	-	-	-	-	*	-	-
Saleh et al., 2012 [36]	-	-	-	-	-	*	-	-
Solini et al., 2019 [46]	-	*	-	-	-	*	-	-
Sturm et al., 2009 [30]	-	-	-	-	-	*	-	-
Tarzia et al., 2017 [35]	-	-	-	-	-	*	-	-
Tromba et al., 2017 [42]	-	-	-	-	-	*	-	-
Tschoner et al., 2013 [31]	-	*	-	-	-	*	-	-
Williams et al., 2005 [49]	-	-	-	-	-	*	-	-
Zhang et al., 2014 [40]	-	-	-	-	-	*	-	-

## Data Availability

This review article does not contain any raw data.

## Supplementary Materials

The following supporting information can be downloaded at: https://www.mdpi.com/article/10.3390/jcm11144054/s1. Summary of search strategy used for this study.
