# Supplementary material for: Effect of Bariatric Surgery on Flow-Mediated Vasodilation as a Measure of Endothelial Function: A Systematic Review and Meta-Analysis
Source: J Clin Med. 2022 Jul 13;11(14):4054. doi: 10.3390/jcm11144054 (PMC9323618; doi:10.3390/jcm11144054)
Supplement: Supplementary file 1 [file jcm-11-04054-s001.zip › jcm-1696173-supplementary.pdf]

Database	Search string	Results	Notes
**Source:**<br><br>PubMed<br><br>(NLM)<br><br>**Coverage/search date:**<br><br>from inception -<br><br>2020-04-14	(("bariatric surgery"[title/abstract] OR gastroplast*[title/abstract] OR "gastric bypass"[title/abstract] OR "Roux-en-Y"[title/abstract] OR "gastric band"[title/abstract] OR "biliopancreatic diversion"[title/abstract] OR gastrectom*[title/abstract] OR "duodenal switch"[title/abstract] OR "gastrointestinal diversion"[title/abstract] OR gastroenterostom*[title/abstract] OR "jejunoileal bypass"[title/abstract] OR "obesity surgery"[title/abstract] OR "weight loss surgery"[title/abstract] OR "weight-loss surgery"[title/abstract] OR "bariatric procedure"[title/abstract] OR "sleeve surgery"[title/abstract] OR "metabolic surgery"[title/abstract]) AND (FMD[title/abstract] OR bFMD[title/abstract] OR fFMD[title/abstract] OR "flow mediated dilation"[title/abstract] OR "flow-mediated dilation"[title/abstract] OR "flow mediated dilatation"[title/abstract] OR "flow-mediated dilatation"[title/abstract] OR "flow mediated vasodilation"[title/abstract] OR "flow-mediated vasodilation"[title/abstract] OR "flow mediated vasodilatation"[title/abstract] OR "flow-mediated vasodilatation"[title/abstract]))	**29**	All search terms are searched in the search fields: “title” and “abstract” (here marked with TI/AB) and in MeSH (when available).<br><br>A filter for English language is applied.
**Source:**<br><br>Scopus<br><br>(Elsevier)<br><br>**Coverage/search date:**<br><br>from inception - 2020-04-14	(TITLE-ABS-KEY: ("bariatric surgery" OR gastroplast* OR "gastric bypass" OR "Roux-en-Y" OR "gastric band" OR "biliopancreatic diversion" OR gastrectom* OR "duodenal switch" OR "gastrointestinal diversion" OR gastroenterostom* OR "jejunoileal bypass" OR "obesity surgery" OR "weight loss surgery" OR "weight-loss surgery" OR "bariatric procedure" OR "sleeve surgery" OR "metabolic surgery") AND (TITLE-ABS-KEY (FMD OR bFMD OR fFMD OR "flow mediated dilation" OR "flow-mediated dilation" OR "flow mediated dilatation" OR "flow-mediated dilatation" OR "flow mediated vasodilation" OR "flow-mediated vasodilation" OR "flow mediated vasodilatation" OR "flow-mediated vasodilatation")	**38**	All search terms are searched in the search fields: “title, “abstract” and “keywords” (here marked with: TITLE-ABS-KEY”).<br><br>No thesaurus or subject headings available.<br><br>A filter for English language is applied.
**Source:**<br><br>Web of Science<br><br>(Core Collection, Clarivate)<br><br>**Coverage/search date:**<br><br>from inception -	(TS=("bariatric surgery" OR gastroplast* OR "gastric bypass" OR "Roux-en-Y" OR "gastric band" OR "biliopancreatic diversion" OR gastrectom* OR "duodenal switch" OR "gastrointestinal diversion" OR gastroenterostom* OR "jejunoileal bypass" OR "obesity surgery" OR "weight loss surgery" OR "weight-loss surgery" OR "bariatric procedure" OR "sleeve surgery" OR "metabolic surgery") AND TS=(FMD OR bFMD OR fFMD OR "flow mediated dilation" OR "flow-mediated dilation" OR "flow mediated dilatation" OR "flow-mediated dilatation" OR "flow mediated vasodilation" OR "flow-mediated vasodilation" OR "flow mediated vasodilatation" OR "flow-mediated vasodilatation"))	**36**	All search terms are searched in the field: “TOPIC” (including title, abstract and author supplied keywords).<br><br>No thesaurus or subject headings available.

2020-04-14			A filter for English language is applied.
**Source:**<br><br>Embase<br><br>(Source: Embase only, Elsevier)<br><br>**Coverage/search date:**<br><br>from inception -<br><br>2020-04-14	("bariatric surgery":ti,ab OR gastroplast*:ti,ab OR "gastric bypass":ti,ab OR "Roux-en-Y":ti,ab OR "gastric band":ti,ab OR "biliopancreatic diversion":ti,ab OR gastrectom*:ti,ab OR "duodenal switch":ti,ab OR "gastrointestinal diversion":ti,ab OR gastroenterostom*:ti,ab OR "jejunoileal bypass":ti,ab OR "obesity surgery":ti,ab OR "weight loss surgery":ti,ab OR "weight-loss surgery":ti,ab OR "bariatric procedure":ti,ab OR "sleeve surgery":ti,ab OR "metabolic surgery":ti,ab) AND (FMD:ti,ab OR bFMD:ti,ab OR fFMD:ti,ab OR "flow mediated dilation":ti,ab OR "flow-mediated dilation":ti,ab OR "flow mediated dilatation":ti,ab OR "flow-mediated dilatation":ti,ab OR "flow mediated vasodilation":ti,ab OR "flow-mediated vasodilation":ti,ab OR "flow mediated vasodilatation":ti,ab OR "flow-mediated vasodilatation":ti,ab)	**48**	All search terms are searched in the fields: “title” and “abstract” (here marked with “:ab,ti”) and in the “thesaurus” (here marked with “/de”) when available.<br><br>Thesaurus (Emtree) variations compared to PubMed’s MeSH are applied as per availability and recommendations Embase.<br><br>A filter for English language is applied.
**Other sources**		13	
**Total no. of records identified**		164	
**Total no. of unique records after de-duplication**		69	
